# Skin Toxicities Associated with Botulin Toxin Injection for Aesthetic Procedures: Data from the European Spontaneous Reporting System

**DOI:** 10.3390/ph16111611

**Published:** 2023-11-15

**Authors:** Maria Maddalena Nicoletti, Antonietta Anatriello, Valerio Liguori, Andrea Cantone, Gabriella di Mauro, Imma Izzo, Nicoletta Lettera, Joao Marcos Della Ragione, Maria Rosaria Campitiello, Vincenzo Cosenza, Cristina Scavone

**Affiliations:** 1 Department of Precision Medicine, University of Campania “Luigi Vanvitelli”, 80138 Naples, Italy; 2 Department of Experimental Medicine, University of Campania “Luigi Vanvitelli”, 80138 Naples, Italy; antoanat98@gmail.com (A.A.); valerio.liguori@unicampania.it (V.L.); andreacantone9800@gmail.com (A.C.); 3 Regional Center of Pharmacovigilance and Pharmacoepidemiology of Campania Region, 80138 Naples, Italy; gabriella.dimauro@unicampania.it (G.d.M.); imma.izzo97@libero.it (I.I.); letteranicoletta@gmail.com (N.L.); jmdellaragione89@gmail.com (J.M.D.R.); 4 UOC Pharmacy, AORN Santobono Pausilipon Children’s Hospital, 80129 Naples, Italy; 5 Department of Obstetrics and Gynaecology and Physiopathology of Human Reproduction, ASL Salerno, 84124 Salerno, Italy; maracampitiello@gmail.com; 6 Department of Environmental, Biological and Pharmaceutical Sciences and Technologies (DiSTABiF), University of Campania “Luigi Vanvitelli”, 80138 Naples, Italy; vincenzocosenza01@gmail.com

**Keywords:** botulin toxin, aesthetic procedures, Eudravigilance, safety, skin toxicities, spontaneous reporting system

## Abstract

Botulinum toxin is a protein deriving from the bacteria *Clostridium botulinum* and it is widely used for the treatment of a variety of muscle hyperactivity syndromes and for cosmetic indications. Having a long-lasting effect, Botulinum toxin type A (BTA) is one of the most botulin toxin products used. Even if BTA has shown benefits in reducing the vertical lines between the eyebrows, Adverse Drug Reactions (ADRs) have been experienced as well, of which the most common ones are headache and drooping eyelids. In addition, since other local and systemic risks have been identified, a non-interventional post-authorization safety study (PASS) has been started. The aim of the present study was to report cases of skin toxicity associated with this drug, considering Individual Case Safety Reports (ICSRs) existing on the Eudravigilance website. Among 1464 ICSRs sent to the EV database, 718 ICSRs, including 5154 PTs, reported BTA as a suspected drug associated with cutaneous toxicity. The majority of patients experiencing BTA-induced skin toxicity were female (92.1%) belonging mostly to the age group of 18–64 years. The most serious criteria, when reported, were “Other Medically Important Condition” and “Caused/prolonged hospitalization”, although the outcome was mainly reported as “Unknown”. The most reported PTs, related to skin disorders, were: “Erythema”, “Rash”, “Pruritus”, “Urticaria”, “Swelling face”, “Brow ptosis”, “Eyelid ptosis”, “Injection site pain”, and “Angioedema”. Considering that in most ICSRs, ADRs related to skin disorders were symptoms of hypersensitivity reactions which in some conditions could be life-threatening, further studies are required to better define the safety profile of BTA used for aesthetic procedures.

## 1. Introduction

Botulinum toxin has been used for more than 20 years to treat many clinical conditions, such as blepharospasm, strabismus, cervical dystonia, migraines, hyperhidrosis, and muscle spasticity [1]. It is a neurotoxin produced by *C. botulinum* that is able to play a role in relaxing muscles by blocking the release of acetylcholine from the nerve synapse. Thus, the muscle does not contract, and the skin is not creased. Complete lack of muscle activity occurs after approximately 5–15 days [2,3]. *C. botulinum* produces seven botulin toxin serotypes (A, B, C, D, E, F, G) [4]. The botulin toxin A (BTA) is the most potent serotype that is available, together with the type B, for the clinical use [5,6]. In 2002, BTA received the approval from the U.S. Food and Drug Administration (FDA) and from the European Medicines Agency (EMA) for cosmetic use [1,7]. Naturally, all botulin serotypes are non-covalently associated with complexing protein, which are instead encoded as nonhemagglutinin and hemagglutinin proteins in two gene clusters on the *C. botulinum* chromosome. Complexing proteins seem to have several functions. For instance, they help in stabilizing the biologic activity of the neurotoxin in vivo and facilitate the adherence to muscle tissue, but also reduce the diffusion of botulinum toxin out of the target tissues, due to the large size of the toxin complex [8,9,10].

BTA is administered through an intramuscular injection at a dosage that depends on the botulinum toxin preparations. Currently, BTA injection is recognized as the most common cosmetic procedure performed worldwide, with estimates of nearly 3 million injections per year [11,12]. As reported in the European Public Assessment Report (EPAR) of a product containing BTA and approved by the EMA, among 540 adults with moderate or severe vertical lines, BTA made the vertical lines between the eyebrows less noticeable compared to placebo (87% of adults who received the drug had either mild or no vertical lines between the eyebrows compared with 4% of patients who received placebo). On the other hand, the most common adverse drug reactions (ADRs) associated with the drug included headache and drooping eyelids. According to data reported in the EPAR, the main identified risks include eyelid ptosis, immunogenicity, distant spread of toxin, development of or exacerbation of neuromuscular disorders, and hypersensitivity reactions (with symptoms that included eyelid edema, injection site pruritus, and influenza-like illness) [2]. To further quantify these safety concerns, the non-interventional post-authorization safety study (PASS) NCT05481931 was initiated [13]. This multicentre observational study is currently enrolling patients to evaluate safety data from approximately 750 patients across 20 sites throughout the United Kingdom and the European Union over an 18-month evaluation period. In addition, as reported by Wollina U et al. [14], local ADRs, including pain, hematoma, ecchymosis, and bruising, are the most common botulin toxin-induced adverse effects. The authors also reported that cooling the skin before and after the BTA injection should be sufficient to prevent them. Similarly, Cohen JL et al. reported that bleeding, swelling, erythema, and pain at the injection sites are the most common side effects of botulin toxin type A [15]. The most common complications are ecchymosis and purpura, which could be minimized by compressing ice on the injection sites before and after the Botox injection [16,17].

Considering the widespread use of BTA for aesthetic purposes, the present study aimed to evaluate the occurrence of skin toxicities associated with this drug by describing data from Individual Case Safety Reports (ICSRs) retrieved from the European spontaneous reporting system (EudraVigilance-EV).

## 2. Results

During the study period, BTA and BTA-haemoagglutinin complex-induced skin disorders were reported in 1437 ICSRs and 352 ICSRs, respectively. After the removal of duplicates (those ICSRs reporting both drugs as suspected), of ICSRs reporting therapeutic indications not related to cosmetic procedures or those not reporting therapeutic indications at all and ICSRs related to pediatric cases, 718 ICSRs, covering 5154 PTs, were included in the final analysis.

As reported in Table 1, the majority of the patients who experienced skin toxicities following BTA and BTA-haemoagglutinin complex injections belonged to the age group 18–64 years (67.0%), and a higher proportion of them were female (92.1%). No substantial difference was found in terms of the primary source country for regulatory purposes, while the majority of ICSRs were reported by Healthcare Professionals (83.9%). The majority of the ICSRs (almost 55%) reported BTA or BTA-haemoagglutinin complex as the only suspected drug and did not report concomitant medications (71.3%) (Table 1).

**Figure 1:**
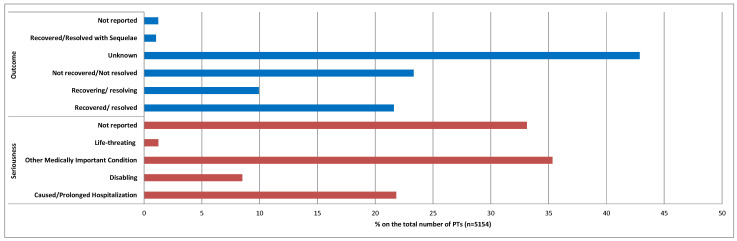
Distribution of Preferred Terms (PTs) by seriousness and outcome degrees.

Apart from 1707 PTs for which the seriousness degree was not defined (33% of the total PTs), the remaining 3447 PTs described ADRs that were classified as serious, mainly as “Other Medically Important Condition” and “Caused/prolonged hospitalization” (Figure 1). In addition, in nine ICSRs (covering a total of 64 PTs) the seriousness criterion was defined as “Life-threating”. These ICSRs mainly described hypersensitivity reactions with local and systemic symptoms occurring in women. For these cases, concomitant and suspected drugs other than BTA/BTA-haemoagglutinin complex were reported in four out of nine ICSRs (Table 2).

The outcome was not known for almost 43% of all reported PTs; it was reported as favorable (recovered/resolved, recovering/resolving) for almost 30% of PTs, while it was reported as unfavorable (not recovered/not resolved, recovered/resolved with sequelae) for almost 25% of PTs (Figure 1).

As shown in Table 3, the following PTs were reported in more than 50 ICSRs: “Off-label use”, “Erythema”, “Headache”, “Rash”, “Pruritus”, “Urticaria”, “Swelling face”, “Brow ptosis”, “Dyspnea”, “Hypersensitivity”, “Dizziness”, “Eyelid ptosis”, “Injection site pain”, “Nausea”, “Fatigue”, “Angioedema”, “Pain”, “Vision blurred”, “Hypoesthesia”, and “Malaise”. All together these PTs accounted for 30.6% of all reported PTs. Furthermore, the following PTs: “Injection site swelling”, “Hyperidrosis”, “Skin tightness”, “Face oedema”, “Facial paralysis”, and “Injection site erythema”, belonging to the SOC “Skin disorders”, were at least reported in 24 ICSRs.

## 3. Discussion

In the countries where BTA is used, there is a general remark that a youthful appearance might correspond to an increased powerful, successful, and happy life [18]. The use of BTA for aesthetic use started in the 1980s, but it was not until 20 years later that the first and most popular product containing the drug was approved by the US and EU regulatory agencies. Data from a recent review [19] highlighted that a shift in botulin utilization has been observed in recent years among younger adults who are much more interested—compared to previous generations—to prevent rather than correct signs of facial aging and to delay the signs of aging before they become prominent, the so-called medical phenomena defined as “prejuvenation” [20]. Prejuvenation has been popular amongst Generation Z since 2000 due to the accelerated advancements in skincare and aesthetics and the concomitant rise of social media as a new source of information. For younger adults, prejuvenation can involve adopting a healthy lifestyle, using skincare products with anti-aging properties, but also considering minimally invasive cosmetic procedures, including BTA injections [21]. The American Society of Dermatologic Surgery confirms an increase of 50%, between 2012 and 2016, in botulin use among patients in their 30s or even younger, estimating that younger adults will be the most significant users of botulin toxin by 2025 [22]. In addition, according to the American Society of Plastic Surgeons, 92% of all cosmetic procedures (including surgical and minimally invasive) carried out in the U.S. in 2020 were done by women with a peak in the age group 40–54 years for which 5.4 million minimally-invasive procedures were registered [23]. Taken together, all these data confirmed findings from our study, showing that BTA-induced ADRs mainly occurred in women belonging to the age group 18–64 years.

As reported by Naumann M et al. [24], the most common botulin toxin A-induced ADRs include local reactions, such as pain, edema, erythema, ecchymosis, and short-term hyperesthesia which is in line with our results showing that these ADRs were reported in almost 300 ICSRs retrieved form the EV in our study. Local events are common, mild, and tend to spontaneously resolve within 24 h [12]. On the other hand, systemic ADRs following botulin toxin A injection commonly include headhache, nausea, fatigue, malaise, flu-like symptoms, and rash [24]. These reactions are the result of the systemic spread of the toxin. Recently, Gostimir M et al. carried out a meta-analysis of randomized controlled trials (RCTs, published until January 2020) to evaluate the safety profile of the drug used for cosmetic indications compared to placebo, with special attention to clinically relevant covariates and their relative impact on safety. The study included 32 RCTs involving 9669 patients. The pooled RR of any treatment-related adverse events occurring after BTA injections compared to placebo injection was 1.53 (95% CI, 1.33–1.77). AEs more likely associated with BTA rather than placebo included eyelid/eyebrow malposition (RR 3.55; *p* < 0.001), facial paresis (RR 2.42; *p* = 0.316), and headache (RR 1.45; *p* = 0.003), while injection site reactions occurred at similar rates in both groups [25]. These results are in line with our findings related to the most common PTs found in the EV database; as a matter of fact, all AEs mentioned in the meta-analysis by Gostimir M et al. were reported in more than 29 ICSRs (in our study headache was reported in 124 ISCRs, brow ptosis and eyelid ptosis—a form of eyelid/eyebrow malposition—were reported in 76 and 60 ICSRs, respectively, and facial paresis in 29 ICSRs). These reactions, especially local ones such as ptosis, represents the consequence of the toxic diffusion across cutaneous tissue, such as in the case of ptosis [26]. This complication can be avoided by injecting the drug into the frontalis keeping a distance of 2–3 cm above the supraorbital margin or 1.5–2 cm above the eyebrow [27,28]. In line with cutaneous PTs commonly reported among ICSRs retrieved in our study, data from a recent literature review suggest that other local common ADRs associated with BTA injections include local edema, erythema, bruising, and pain at the injection and adjacent sites [29]. Authors of the review reported that in order to prevent local bruising, the use of a small gauge needle might be helpful as well as the use of ice that can also reduce the pain.

Regarding the seriousness and outcome degrees, we found that almost 67% of ICSRs described serious cutaneous ADRs with a favorable outcome in almost 30% of cases. Specifically, among serious cases, there were nine ICSRs describing life-threating ADRs, the majority of which reported hypersensitivity reactions, including three cases of anaphylaxis. Coté TR et al. evaluated the ICSRs related to botulin toxin A sent to the FDA MedWatch system until 2003. Authors reviewed 1437 ICSRs, highlighting an increase in annual reporting of serious ADRs from two in 1991 to 41 in 2002, correlating with the increase in annual sales during the same years. As detected in our study, patients who experienced ADRs were predominantly female with a median age of 50 years. Among serious cases, the most commonly reported ADRs were headache, focal facial paralysis, muscle weakness, dysphagia, flu-like syndromes, and allergic reactions [30]. Authors concluded that serious ADRs are not so commonly reported during the cosmetic use of the drug and that the majority of these events were previously recognized during pre-marketing clinical trials. However, despite this encouraging data, many publications have documented the occurrence of serious immunologically-mediated ADRs, including anaphylactic reactions. For instance, Moon IJ et al. reported the case of a 35-year-old woman who experienced anaphylaxis about 5 min after the second intramuscular injection of BTA. To manage the reaction intramuscular epinephrine was administered [31]. Another case was reported by Li M et al. [32] who described the occurrence of a fatal anaphylaxis that, according to medical records, autopsy, and laboratory findings, was determined by the Botox-lidocaine mixture. Cases of anaphylactic shock to lidocaine have been previously reported [33,34] and among the life-threating cases described in our study there was an ICSR describing the occurrence of angioedema in a woman receiving BTA, tozinameran, hyaluronic acid, and lidocaine. Thus, although local allergic reactions (presenting with edema, erythema or redness) seem to be the most common BTA-induced ADRs, a risk of diffuse erythema, urticaria, and anaphylactic shock cannot be excluded [35]. Systemic symptoms are often serious, but they more commonly occur when BTA is used for non-aesthetic procedures and a higher median botulin toxin dose is used (normally four times higher) [29]. In order to reduce the risk of allergic reactions, including anaphylaxis, it is essential to thoroughly review patients’ medical history for any previous similar reactions as well as medical conditions that could predispose them to this risk.

However, as reported by Pickett A, the dosage of BTA normally used for intramuscular injection is not expected to result in the systemic circulation of BTA, which represents one of the major benefits of BTA, as it produces a localized clinical effect [36].

The safety analysis carried out for this study, which was based on a pharmacovigilance database, has both conventional strengths and limitations. Safety data deriving from the spontaneous reporting system reflect the real-world experience of drugs, including drug use patterns that cannot be examined in clinical studies due to ethical considerations. In addition, this kind of data represents a valuable source to identify uncommon ADRs and to detect medication errors that are not visible in pre-marketing clinical studies. EudraVigilance, the largest pharmacovigilance database, also gathers heterogeneous data from many demographics and nations [37,38,39]. Considering that the data analyzed are related to ICSRs, gathered during routine clinical practice, and thus referred to medications used in real-world settings, the data we have analyzed are very important. Indeed, despite randomized controlled trials represents the gold standard for assessing the efficacy and safety of treatments, their inherent limitations limit the applicability of their findings to the general population. In this context, the collection and analysis of efficacy and safety data from real-life conditions help in overcoming this gap.

On the other hand, the spontaneous reporting system has intrinsic limitations such as the low reporting rate of ADRs (under-reporting) and the lack of data quality (data reported in ICSRs are often incomplete or imprecise). As a matter of fact, many ICSRs retrieved for this study lacked information on the age group (not specified in 27% of the total retrieved ICSRs) and seriousness degree (missing in 33.1% of ICSRs). Being spontaneous, ICSRs often lack patients’ demographic and clinical data, which makes not possible to draw a causal relationship between drugs and ADRs and the proper evaluation of each case becomes difficult. In line with this, data reported by the WHO Uppsala Monitoring Centre revealed that in 2014 only 13% of ICSRs had a good degree of information completion although, according to Good Pharmacovigilance Practices (GVPs) [40], four criteria are mandatory for ICSRs validation [an identifiable reporter, an identifiable patient (initials, date of birth, gender or age), one or more suspected drugs, and one or more suspected ADRs]. In addition to these mandatory data, other criteria are desirable to have a well-documented report, including basic medical condition, comorbidities, concomitant medication, ADR’s management and outcome, data on dechallenge, and dechallenge [41,42,43]. This unreported or missing information could have influenced our analysis. Furthermore, in this study the majority of ICSRs were reported by healthcare professionals that undoubtedly represent a valuable source for collecting safety data during the post-marketing phase. However, these reporters are more incline to report serious ADRs [44,45], as happened in this study. Thus, a reporting bias cannot be excluded. In conclusion, due to the limitations affecting this study, it is not simple to interpret and evaluate these data from a clinical point of view and further studies characterizing the cutaneous safety profile of BTA products are strongly needed.

## 4. Material and Methods

### 4.1. Data Source

Data on ICSRs were retrieved from the EV website (publicly accessible at www.adrreports.eu), the European spontaneous reporting system that allows the collection and analyses of ICSRs related to medicines authorized or being studied in clinical trials in the European Economic Area (EEA) and that is managed by the EMA.

By using the line listing function of the EV website, ICSRs reporting BTA or BTA-haemoagglutinin complex as suspected drugs and used for cosmetic purposes and cases belonging to the Reaction group “Skin disorders” were retrieved from 2002 (the date of marketing authorization granted by the EMA) to 1 May 2023.

ICSRs were included in the analysis only if the therapeutic indication of the suspected drug(s) included “Skin wrinkles”, “Cosmetic procedure” or “Facial asymmetry”. ICSRs reporting therapeutic indications not related to cosmetic procedures (including cases where the therapeutic indication was not specified) were excluded. Pediatric cases were excluded as well.

### 4.2. Descriptive Analyses

Information on patient characteristics [age group (18–64 years and 65–85 years) and sex], adverse event (type, outcome and seriousness), primary source qualification, primary source country for regulatory purposes, number of suspected drugs other than BTA and BTA-haemoagglutinin complex, and number of concomitant drugs was provided for all ICSRs.

According with the International Council on Harmonization E2D guidelines, a case is defined as “serious” if it is life-threatening, results in death, requires or prolongs a hospitalization, results in persistent or significant disability/incapacity, determines a congenital anomaly/birth defect, or results in some other clinically important conditions [46]. The outcome was classified as favorable (“Recovered/Resolved” and “Recovering/Resolving”), unfavorable (“Recovered/Resolved with Sequelae”, “Not Recovered/Not Resolved”, “Fatal”) and not reported (“Unknown”). The outcome with the lower level of resolution was chosen for classification whether an ICSR reported two or more PTs with different outcomes. ICSRs were classified as fatal if death occurred.

Data were analyzed using Microsoft Office Excel 2010 program.

### 4.3. Ethical Standards

Safety data extracted from the spontaneous reporting system comply with ethical standards and are anonymous. Therefore, no further ethical measures were enforced.

## 5. Conclusions

We carried out a pharmacovigilance study using data from the EV database with the aim to describe the main characteristics of cutaneous ADRs associated with BTA and BTA-haemoagglutinin complex injections. During almost 20 years of drug utilization, 718 ICSRs related to BTA or BTA-haemoagglutinin used for cosmetic purposes and describing the occurrence of skin toxicities were reported to the EV database. In many ICSRs, the BTA-induced skin toxicity represented a symptom of an hypersensitivity reaction that, although rare, may vary from mild local symptoms to life-threatening systemic anaphylaxis. Thus, a better characterization of anaphylaxis cases associated with intramuscular injections of BTA through a detailed analysis of ICSRs from the EV is highly recommended. In general, considering the increasing use of BTA injections for aesthetic procedures and given the fact that in some cases BTA-induced ADRs can be serious, it is essential for clinicians to closely monitor patients during and after the procedure. On the other hand, considering that the use of aesthetic medicine goes along with the rise of social media’s spread of information, especially among younger adults, it is desirable that patients consult qualified healthcare professionals, including dermatologists, who can provide them with necessary information and guidance, helping them understand the benefits, the risks, and potential ADRs associated with BTA injections.

## Figures and Tables

**Table 1:** Demographic characteristics and distribution for seriousness, outcomes, primary source, primary source country for regulatory purposes, number of suspected drugs other than botulin toxin type A, and number of concomitant drugs of Individual Case Safety Reports (ICSRs) reporting at least one event related to the SOC “Skin disorders” and having botulin toxin type A and botulin toxin type A haemoagglutinin complex as the suspected drugs among those reported in the Eudravigilance database from the date of marketing authorization to 1 May 2023.

Variable	Level	All ICSRs (*n* = 718), *n* (%)
Age group	18–64 years	479 (67)
	65–85 years	44 (6)
	Not specified	195 (27)
Gender	F	662 (92.1)
	M	40 (5.6)
	Missing	16 (2.3)
Primary Source Qualification	Healthcare Professional	602 (83.9)
	Non-Healthcare Professional	115 (16.0)
	Not specified	1 (0.1)
Primary Source Country for Regulatory Purposes	European Economic Area	378 (52.6)
	Non-European Economic Area	340 (47.4)
Suspected drug(s) other than botulin toxin type A	0	390 (54.3)
	1	164 (22.8)
	2	117 (16.4)
	3	27 (3.8)
	4	20 (2.8)
Concomitant drug(s)	0	512 (71.3)
	1	92 (12.8)
	2	49 (6.8)
	3	25 (3.5)
	4	16 (2.2)
	≥5	24 (3.5)

**Table 2:** ICSRs having botulin toxin type A or botulin toxin type A haemoagglutinin complex as suspected drug and describing Life-threatening ADRs.

Case No.	Age Group	Sex	Preferred Terms (PTs)	Suspect Drug(s) Other Than Botulin Toxin Type A	Concomitant Medication
1	NA	F	Anaphylactic reaction, Dyspnea, Pruritus, Swelling face, Swollen tongue	-	-
2	18–64 Years	F	Anaphylactic reaction, Erythema, Rash pruritic	-	-
3	NA	F	Anaphylactic shock, Chest discomfort, Facial paresis, Hypersensitivity, Nausea, Seborrhea	-	Levothyroxine
4	18–64 Years	F	Acne, Asthenia, Coma, Dyspnea, Erythema, Fatigue, Hemorrhage, Influenza like illness, Off-label use, Pneumonia, Pseudomonas infection, Staphylococcal infection, White blood cell count abnormal	Hyaluronic acid	Alprazolam, albutamol
5	18–64 Years	F	Blood pressure increased, Erythema, Histamine level increased, Hypotension, Localized edema, Tachycardia, Urticaria	-	-
6	18–64 Years	F	Angioedema	Tozinameran, Hyaluronic Acid, Lidocaine	Perindopril Tert-Butylamine, Indapamide
7	18–64 Years	F	Anxiety, Dyspnea, Eye pain, Eye pruritus, Eye swelling, Headache, Hypersensitivity, Incorrect route of product administration, Lacrimation increased, Malaise, Paresthesia oral, Rash, Rash vesicular, Skin discoloration, Urticaria, Vision blurred, Wheezing	-	-
8	18–64 Years	F	Angioedema, Injection site hematoma, Injection site hemorrhage, Injection site swelling, Off label use	-	Lisinopril
9	18–64 Years	F	Angioedema, Face edema	-	-

NA: Not Available.

**Table 3:** List of Preferred Terms (PTs) reported in retrieved Individual Case Safety Reports (ICSRs).

List of PTs Reported in Retrieved ICSRs	
	*N* (%)
Off-label use	179 (3.5%)
Erythema	128 (2.5%)
Headache	124 (2.4%)
Rash	118 (2.3%)
Pruritus	103 (2.0%)
Urticaria	85 (1.6%)
Swelling face	80 (1.5%)
Brow ptosis	76 (1.5%)
Dyspnea	76 (1.5%)
Hypersensitivity	73 (1.4%)
Dizziness	63 (1.2%)
Eyelid ptosis	60 (1.2%)
Injection site pain	58 (1.1%)
Nausea	55 (1.1%)
Fatigue	54 (1.0%)
Angioedema	54 (1.0%)
Pain	53 (1.0%)
Vision blurred	52 (1.0%)
Hypoesthesia	50 (0.9%)
Malaise	50 (0.9%)
Paresthesia	49 (0.9%)
Muscular weakness	48 (0.9%)
Anxiety	45 (0.9%)
Swelling	43 (0.8%)
Product preparation error	43 (0.8%)
Dysphagia	42 (0.8%)
Drug ineffective	42 (0.8%)
Injection site swelling	39 (0.8%)
Asthenia	38 (0.7%)
Hyperhidrosis	38 (0.7%)
Skin tightness	34 (0.7%)
Eye swelling	33 (0.6%)
Insomnia	32 (0.6%)
Palpitations	32 (0.6%)
Dry mouth	30 (0.6%)
Facial paresis	29 (0.6%)
Influenza like illness	28 (0.5%)
Face oedema	28 (0.5%)
Visual impairment	28 (0.5%)
Dry eye	27 (0.5%)
Facial pain	27 (0.5%)
Tremor	27 (0.5%)
Vomiting	27 (0.5%)
Eye pain	26 (0.5%)
Swelling of eyelid	26 (0.5%)
Diarrhoea	26 (0.5%)
Alopecia	25 (0.5%)
Facial paralysis	24 (0.5%)
Injection site erythema	24 (0.5%)
Feeling abnormal	23 (0.4%)
Neck pain	22 (0.4%)
Pyrexia	22 (0.4%)
Migraine	21 (0.4%)
Periorbital swelling	21 (0.4%)
Skin disorder	20 (0.4%)
Muscle spasms	20 (0.4%)
Skin wrinkling	20 (0.4%)
Arthralgia	19 (0.4%)
Skin burning sensation	19 (0.4%)
Weight decreased	19 (0.4%)
Feeling hot	19 (0.4%)
Overdose	19 (0.4%)
Burning sensation	17 (0.3%)
Botulism	17 (0.3%)
Depression	16 (0.3%)
Dry skin	16 (0.3%)
Head discomfort	16 (0.3%)
Swollen tongue	16 (0.3%)
Diplopia	16 (0.3%)
Skin discolouration	16 (0.3%)
Musculoskeletal stiffness	15 (0.3%)
Myalgia	15 (0.3%)
Eye irritation	15 (0.3%)
Muscle twitching	15 (0.3%)
Contusion	15 (0.3%)
Drug hypersensitivity	15 (0.3%)
Acne	15 (0.3%)
Ocular hyperaemia	14 (0.3%)
Chest pain	14 (0.3%)
Photophobia	14 (0.3%)
Dysphonia	13 (0.2%)
Oedema	13 (0.2%)
Rash erythematous	13 (0.2%)
Pain in extremity	13 (0.2%)
Discomfort	13 (0.2%)
Abdominal pain upper	13 (0.2%)
Facial asymmetry	13 (0.2%)
Dysarthria	12 (0.2%)
Syncope	12 (0.2%)
Lip swelling	12 (0.2%)
Injection site pruritus	12 (0.2%)
Throat tightness	12 (0.2%)
Photosensitivity reaction	12 (0.2%)
Tachycardia	12 (0.2%)
Papule	12 (0.2%)
Back pain	11 (0.2%)
Therapeutic response decreased	11 (0.2%)
Confusional state	11 (0.2%)
Night sweats	11 (0.2%)
Flushing	11 (0.2%)
Hypoaesthesia oral	11 (0.2%)
Chest discomfort	11 (0.2%)
Inflammation	11 (0.2%)
Intentional product use issue	11 (0.2%)
Neuralgia	10 (0.2%)
Chills	10 (0.2%)
Skin lesion	10 (0.2%)
Ear discomfort	10 (0.2%)
Tinnitus	10 (0.2%)
Loss of consciousness	10 (0.2%)
Skin exfoliation	10 (0.2%)
Madarosis	10 (0.2%)
Injection site mass	10 (0.2%)
Lymphadenopathy	10 (0.2%)
Skin mass	10 (0.2%)
Incorrect route of product administration	10 (0.2%)
Nodule	10 (0.2%)
Condition aggravated	10 (0.2%)
Neuromuscular toxicity	10 (0.2%)
Other PTs	1626 (31.5%)
Total PTs recorded in 718 retrieved ICSRs	5154 (100%)

The total number of PTs (*n* = 5154) exceeds the total number of ICSRs (*n* = 718) since more than one PT can be reported in one single report.

## Data Availability

Data are contained within the article.
